# Treatment Persistence After Major Cardiovascular Events in Psoriasis: A Retrospective Cohort Study Comparing Biologic, Systemic, and Phototherapy

**DOI:** 10.1111/1346-8138.17949

**Published:** 2025-09-08

**Authors:** Hyun Keun Ahn, Sohee Oh, Hyun‐Sun Yoon

**Affiliations:** ^1^ Department of Dermatology Seoul National University Hospital Seoul Korea; ^2^ Medical Research Collaborating Center SMG‐SNU Boramae Medical Center Seoul Korea; ^3^ Department of Dermatology SMG‐SNU Boramae Medical Center Seoul Korea; ^4^ Department of Dermatology Seoul National University College of Medicine Seoul Korea

**Keywords:** biological products, major adverse cardiac events, physicians, practice pattern, psoriasis

## Abstract

Patients with psoriasis experience significantly higher cardiovascular morbidity compared to the general population. Although certain psoriasis treatments may confer cardioprotective effects, current clinical guidelines addressing treatment continuation following major adverse cardiovascular events (MACE) are lacking. Therefore, we conducted a retrospective cohort study using Korean health insurance data (January 2008–October 2021) to examine treatment modifications after MACE in patients with psoriasis. Patients were categorized based on treatment type: biologics, non‐biologic systemic agents (cyclosporine or methotrexate), and phototherapy. Treatment persistence within 3 months after MACE was assessed using multivariable logistic regression, adjusted for demographic and clinical factors. Subgroup analyses were conducted based on previous MACE history. This retrospective cohort study included 1012 patients with MACE history and 2981 without. Biologic therapies demonstrated significantly superior treatment persistence (79.7%) compared to non‐biologic systemic therapies (46.9%) and phototherapy (47.0%). Switching therapies was uncommon across all treatment groups (0.8%–1.9%). Multivariable logistic regression analysis confirmed that biologics were associated with greater treatment persistence than cyclosporine (odds ratio [OR], 4.854; 95% confidence interval [CI], 2.476–9.516), methotrexate (OR, 3.616; 95% CI, 1.760–7.431), and phototherapy (OR, 4.556; 95% CI, 2.340–8.873). These findings remained consistent in both the new‐onset and recurrent MACE subgroups. No significant difference in treatment persistence was observed between the non‐biologic systemic therapy and phototherapy groups. In conclusion, patients receiving biologic therapies were more likely to continue treatment after a cardiovascular event compared to those receiving other therapies. These findings suggest that biologics are perceived as safer options in this high‐risk population and underscore the need for clear, evidence‐based guidance on psoriasis treatment following cardiovascular events.

## 1 Introduction

Psoriasis and psoriatic arthritis (PsA) are chronic inflammatory conditions associated with a significantly increased risk of cardiovascular disease (CVD) [1, 2, 3, 4]. This elevated CVD risk is attributable not only to a higher prevalence of conventional CVD risk factors, such as obesity, dyslipidemia, hypertension, and diabetes mellitus, but also to chronic systemic inflammation inherent to these conditions. The persistent inflammatory state contributes to endothelial dysfunction and accelerates atherogenesis [5, 6].

Therapeutic interventions for psoriasis exert heterogeneous effects on the cardiovascular risk. Some agents potentially exacerbate this risk through adverse metabolic effects, while others may mitigate the risk by attenuating systemic inflammation. Methotrexate [7] and tumor necrosis factor‐alpha (TNF‐α) inhibitors [8, 9, 10] represent well‐recognized agents associated with reduced cardiovascular risk in patients with psoriasis, PsA, and rheumatoid arthritis. A recent retrospective cohort study demonstrated that both TNF‐α inhibitors and non‐TNF‐α biologics (interleukin [IL]‐12/23, IL‐17, and IL‐23 inhibitors) are associated with a reduced risk of new‐onset and recurrent major adverse cardiovascular events (MACE) in patients with psoriasis [11]. These cardioprotective effects are believed to be mediated by the reduction in systemic inflammation achieved by these agents [12, 13, 14].

Although previous studies have demonstrated the potential cardiovascular benefits of systemic anti‐inflammatory treatments for psoriasis, evidence remains limited regarding the optimal approach for continuing or modifying such therapies following the occurrence of MACE. Given that patients with long‐standing or severe psoriasis are at particularly high risk for cardiovascular events [15, 16], those with a history of MACE may experience more aggressive clinical courses and increased susceptibility to subsequent CVD events. Despite potential therapeutic adjustments following cardiovascular events in real‐world practice, systematic evaluation of these clinical decisions remains insufficient, limiting physicians' ability to make informed treatment decisions and potentially compromising optimal care for this high‐risk patient subgroup.

This population‐based nationwide study aimed to evaluate how the prescription patterns of biologics, non‐biologic systemic agents, and phototherapy are altered following the occurrence of MACE in patients with psoriasis.

## 2 Methods

This study utilized claims data from the Health Insurance Review and Assessment Service (HIRA) in Korea, covering the period from January 1, 2008, to October 31, 2021 (HIRA research data M20220616004). HIRA data represent the National Health Insurance (NHI) system and include nationwide healthcare claims submitted for reimbursement across the Korean population [17].

The study population comprised individuals with at least two outpatient visits or one inpatient admission with a principal diagnostic code for plaque psoriasis (L40*, excluding L40.1, L40.2, and L40.3) or PsA (M07.3, L40.5), according to the International Classification of Diseases, 10th Revision (ICD‐10) diagnostic code.

Each patient was classified into one of the following three treatment groups based on the type of treatment they received for psoriasis: (1) biologic group, including all patients treated with biologics, regardless of concurrent or previous use of non‐biologic agents or phototherapy; (2) non‐biologic systemic group, comprising biologic‐naïve patients treated with cyclosporine or methotrexate, regardless of concurrent or previous use of phototherapy; and (3) phototherapy group, including patients treated with phototherapy alone, without exposure to biologic or non‐biologic systemic agents. The index date was defined as the initiation date of the corresponding treatment in each group, provided that it followed the first claim with a principal diagnosis code for plaque psoriasis.

Patients were excluded from the analysis if they met any of the following criteria: a diagnosis of PsA without psoriasis; absence of treatment history; initiation of the same treatment for non‐psoriasis‐related indications before the index date; use of systemic agents other than cyclosporine and methotrexate; or a single visit per treatment episode.

Patients who experienced MACE after the index date were included in the final analysis. MACE was defined as hospitalization with the principal diagnostic code for acute myocardial infarction, ischemic or hemorrhagic stroke, cardiac arrest, unstable angina, and heart failure [11, 18]. Patients were considered to have a previous history of MACE if they had received a principal diagnostic code for MACE before the index date. New‐onset MACEs were defined as those occurring in patients without a history of MACE, while recurrent MACEs were defined as those occurring in patients with a history of MACE. This study was exempted from Institutional Review Board approval (IRB no. 07‐2022‐24).

### 2.1 Statistical Analysis

We categorized the patients based on their history of MACE (present or absent), and subsequent analyses were conducted accordingly. Categorical variables are presented as frequencies (%), while continuous variables are presented as means ± standard deviations (SDs). Intergroup comparisons were performed using the Student's *t*‐test for continuous variables and the chi‐square test or Fisher's exact test for categorical variables.

To identify patients actively receiving treatment at the time of MACE, we included only those with treatment records within 3 months (91 days) preceding the event. Treatment changes were assessed over 3 months following MACE. The maximum dosing interval for biologic agents and the typical maximum prescription duration for immunosuppressive therapies under the reimbursement criteria was 12 weeks (84 days). To allow for potential delays of approximately 1 week due to changes in visit schedules, the absence of claims data within 3 months after MACE was operationally defined as treatment discontinuation.

The treatment status after MACE was classified as maintained, switched, or discontinued. “Maintained” referred to patients who continued the same treatment they had received prior to MACE within 3 months following the event. “Switched” was defined as the initiation of a different treatment modality within that period. “Discontinued” referred to the cessation of previous treatment without subsequent systemic therapy or phototherapy.

To evaluate treatment persistence after MACE, we calculated the odds ratios (ORs) and 95% confidence intervals (CIs) for each pairwise comparison between the treatment groups using multivariable logistic regression. The Bonferroni correction was applied to account for multiple comparisons. Confounding factors were selected based on clinical relevance and prior literature, and adjustments were made for age, sex, insurance type, concomitant PsA, type of medical center, and number of cardiovascular risk factors.

Subgroup analyses were conducted using two subgroups, new‐onset and recurrent MACE groups, which were categorized based on previous history of MACE.

Analyses were performed using SAS Enterprise Guide Version 7.1 (SAS Institute Inc., Cary, NC, USA) and R software (version 3.5.1; R Foundation for Statistical Computing, Vienna, Austria). All statistical tests were two‐sided, and statistical significance was set at *p* < 0.05.

## 3 Results

### 3.1 Demographic and Clinical Characteristics of Patients

**FIGURE 1:**
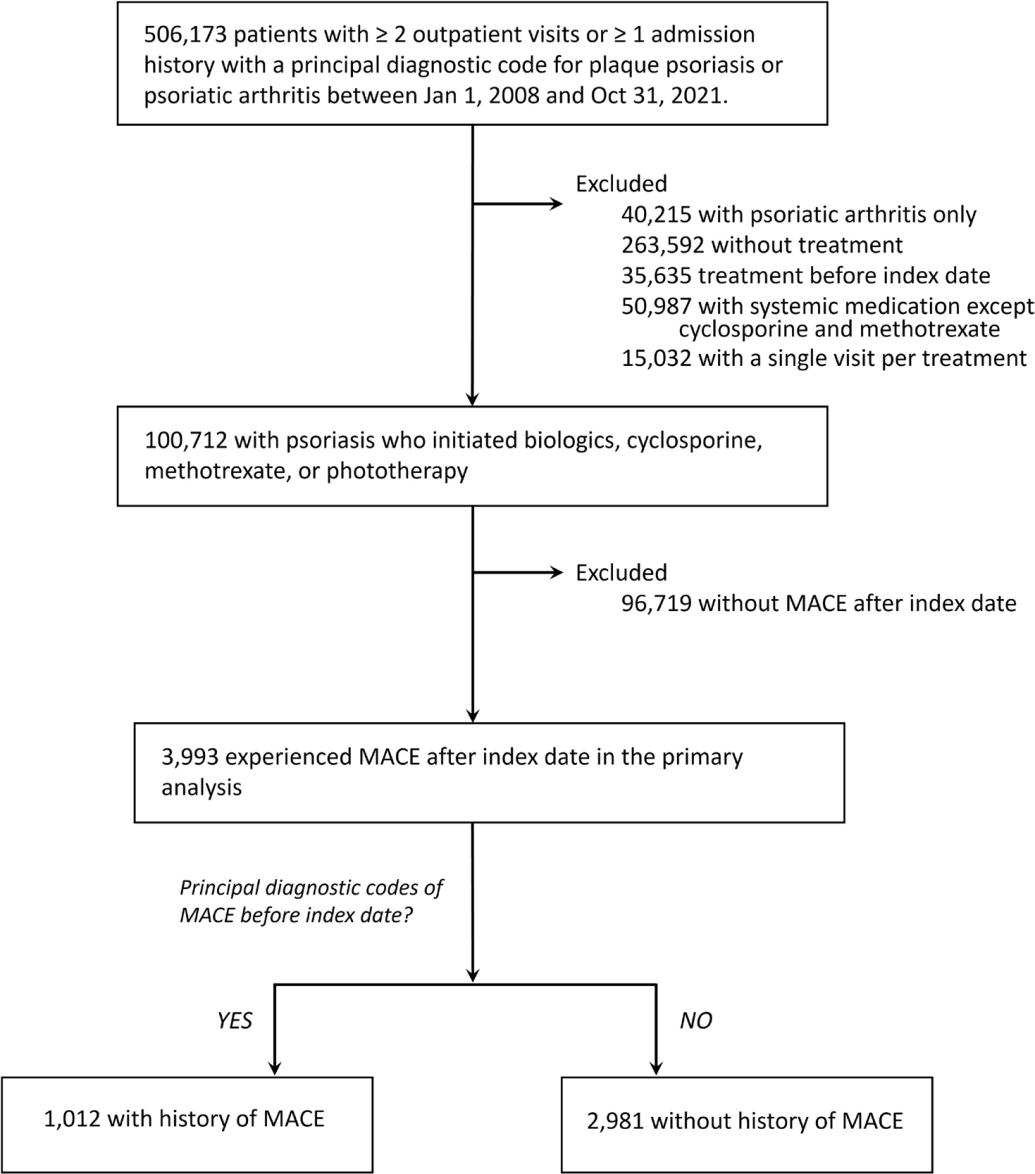
Flow chart of study population selection. ICD‐10, International Classification of Diseases, Tenth Revision; MACE, major adverse cardiovascular event.

Between January 1, 2008, and October 31, 2021, a total of 3993 patients with psoriasis (with or without concomitant PsA) who experienced MACE after the index date were identified (Figure 1). Among them, 2981 patients (74.7%) had no history of MACE prior to treatment initiation (new‐onset MACE), while 1012 patients (25.3%) had a history of MACE before treatment initiation (recurrent MACE).

Demographic and clinical characteristics at the time of MACE after treatment initiation are summarized in Table 1. Most patients were men (*n* = 2788; 72.3%), with a mean age of 64.9 years (SD 13.1 years). Most patients were diagnosed with psoriasis without concomitant PsA (*n* = 3874; 97.0%). The most common comorbidities were hypertension (*n* = 2214; 55.4%), diabetes mellitus (*n* = 1445; 36.2%), and hepatic disease (*n* = 775; 19.4%). Following treatment initiation (index date), the most frequently observed MACE were stroke (*n* = 2163; 54.2%), unstable angina (*n* = 727; 18.2%), and acute myocardial infarction (*n* = 694; 17.4%). Compared to those with new‐onset MACE, patients with recurrent MACE were older, had a higher prevalence of comorbidities, and were more likely to have three or more cardiovascular risk factors at the time of MACE.

**TABLE 1:** Clinical characteristics of the study population at the time of MACE after treatment initiation.

	New‐onset MACE	Recurrent MACE	Overall	*p*
	(*N* = 2981)	(*N* = 1012)	(*N* = 3993)	
Age (years), mean (SD)	63.3 ± 13.2	69.7 ± 11.3	64.9 ± 13.1	< 0.001
> 50 years, *n* (%)	2462 (82.6%)	955 (94.4%)	3417 (85.6%)	< 0.001
Male sex, *n* (%)	2162 (72.5%)	726 (71.7%)	2888 (72.3%)	0.629
Health insurance type				
Health insurance	2722 (91.3%)	860 (85.0%)	3582 (89.7%)	< 0.001
Medical aid	259 (8.7%)	152 (15.0%)	411 (10.3%)	
Diagnosis, *n* (%)				
Psoriasis alone	2884 (96.7%)	990 (97.8%)	3874 (97.0%)	0.081
Psoriasis + psoriatic arthritis	97 (3.3%)	22 (2.2%)	119 (3.0%)	
Type of medical center, *n* (%)				
Tertiary referral hospital	1162 (40.0%)	371 (36.7%)	1533 (38.4%)	< 0.001
General hospital	1744 (58.5%)	577 (57.0%)	2321 (58.1%)	
Private clinic and others	75 (2.5%)	64 (6.3%)	139 (3.5%)	
Comorbidities, *n* (%)				
Hypertension	1565 (52.5%)	649 (64.1%)	2214 (55.4%)	< 0.001
Diabetes mellitus	968 (32.5%)	477 (47.1%)	1445 (36.2%)	< 0.001
Dyslipidemia	513 (17.2%)	151 (14.9%)	664 (16.6%)	0.091
Obesity	4 (0.1%)	0 (0.0%)	4 (0.1%)	0.578*
Renal disease	177 (5.9%)	147 (14.5%)	324 (8.1%)	< 0.001
Hepatic disease	589 (19.8%)	186 (18.4%)	775 (19.4%)	0.338
*N* of cardiovascular risk factors, *n* (%)^a^				
0	300 (10.1%)	20 (2.0%)	320 (8.0%)	< 0.001
1	770 (25.8%)	211 (20.8%)	981 (24.6%)	
2	1120 (37.6%)	388 (38.3%)	1508 (37.8%)	
≥ 3	791 (26.5%)	393 (38.8%)	1184 (29.7%)	
Type of major cardiovascular events after index date, *n* (%)				
Acute myocardial infarction	553 (18.6%)	141 (13.9%)	694 (17.4%)	0.001
Unstable angina	508 (17.0%)	219 (21.6%)	727 (18.2%)	0.001
Heart failure	268 (9.0%)	156 (15.4%)	424 (10.6%)	< 0.001
Stroke	1664 (55.8%)	499 (49.3%)	2163 (54.2%)	< 0.001

*Note:* *p*‐values for categorical variables were calculated using the chi‐square test unless otherwise specified.

Abbreviations: MACE, major adverse cardiovascular event; SD, standard deviation.

* *p*‐value for obesity was calculated using Fisher's exact test.

^a^ Cardiovascular risk factors included age > 50 years, hypertension, diabetes mellitus, dyslipidemia, and obesity.

### 3.2 Treatment Persistence and Modifications After MACE

Treatment modifications within 3 months after MACE were evaluated in 1169 patients with a documented treatment history during the 3 months preceding MACE (Table 2). Among patients receiving biologics before MACE, 79.7% maintained the same regimen, compared to 46.9% and 47.0% of those receiving non‐biologic systemic agents and phototherapy, respectively. Discontinuation rates were lower among patients receiving biologic agents (19.5%) than among those receiving non‐biologic systemic agents (46.7%) and phototherapy (51.1%). Treatment switching was infrequent in all groups, particularly among those receiving biologics (0.8%).

**TABLE 2:** Changes in treatment within 3 months after MACE.

History of MACE	Treatment in 3 months before MACE	Maintained	Switched	Discontinued
All MACE	Biologics	106 (79.7%)	1 (0.8%)	26 (19.5%)
	Non‐biologic systemic	269 (46.9%)	37 (6.4%)	268 (46.7%)
	CSA	178 (44.8%)	22 (5.5%)	197 (49.6%)
	MTX	91 (51.4%)	15 (8.5%)	71 (40.1%)
	Phototherapy	217 (47.0%)	9 (1.9%)	236 (51.1%)
New‐onset MACE	Biologics	84 (78.5%)	1 (0.9%)	22 (20.6%)
	Non‐biologic systemic	178 (44.6%)	30 (7.5%)	191 (47.9%)
	CSA	114 (43.0%)	17 (6.4%)	134 (50.6%)
	MTX	64 (47.8%)	13 (9.7%)	57 (42.5%)
	Phototherapy	157 (47.4%)	7 (2.1%)	167 (50.5%)
Recurrent MACE	Biologics	22 (84.6%)	0 (0%)	4 (15.4%)
	Non‐biologic systemic	91 (52.0%)	7 (4.0%)	77 (44.0%)
	CSA	64 (48.5%)	5 (3.8%)	63 (47.7%)
	MTX	27 (62.8%)	2 (4.7%)	14 (32.6%)
	Phototherapy	60 (45.8%)	2 (1.5%)	69 (52.7%)

Abbreviations: CSA, cyclosporine; MACE, major adverse cardiovascular event; MTX, methotrexate.

This trend was consistent when stratified according to MACE history. In the new‐onset MACE group, biologic therapy was maintained in 78.5% of patients, while 44.6% and 47.4% of patients were maintained on non‐biologic systemic therapy and phototherapy, respectively. In the recurrent MACE group, treatment persistence was highest among biologic users (84.6%), followed by those receiving non‐biologic systemic therapy (52.0%) and phototherapy (45.8%). No notable difference in treatment prescription patterns was observed when analyzing nonbiologic by cyclosporine and methotrexate separately.

Logistic regression analysis was used to adjust for age, sex, insurance type, concomitant PsA, type of medical center, and number of cardiovascular risk factors and showed that biologic therapy was significantly associated with greater treatment persistence compared to cyclosporine (OR, 4.854; 95% CI, 2.476–9.516; *p* < 0.001), methotrexate (OR, 3.616; 95% CI, 1.760–7.431; *p* < 0.001), and phototherapy (OR, 4.556; 95% CI, 2.340–8.873; *p* < 0.001; Table 3). Similar trends were observed in subgroup analyses. Biologic therapy was consistently associated with greater treatment persistence than non‐biologic systemic agents or phototherapy in the new‐onset group. In the recurrent MACE subgroup, biologic therapy was associated with higher treatment persistence than cyclosporine and phototherapy, but not significantly higher than methotrexate. No significant difference in treatment persistence was observed between non‐biologic systemic therapy and phototherapy, irrespective of MACE history.

**TABLE 3:** Treatment persistence after MACE according to the treatment groups.

History of MACE	Comparison	Adjusted odds ratio	95% confidence interval^a^	*p* ^a^
All MACE	Biologics vs. CSA	4.854	2.476, 9.516	< 0.001
	Biologic vs. MTX	3.616	1.760, 7.431	< 0.001
	Biologics vs. Phototherapy	4.556	2.340, 8.873	< 0.001
	CSA vs. Phototherapy	0.939	0.650, 1.356	1.000
	MTX vs. Phototherapy	1.260	0.778, 2.040	1.000
	CSA vs. MTX	0.745	0.456, 1.216	0.677
New‐onset MACE	Biologics vs. CSA	4.880	2.285, 10.423	< 0.001
	Biologic vs. MTX	3.887	1.740, 8.683	< 0.001
	Biologics vs. Phototherapy	4.200	1.991, 8.861	< 0.001
	CSA vs. Phototherapy	0.861	0.550, 1.347	1.000
	MTX vs. Phototherapy	1.080	0.614, 1.901	1.000
	CSA vs. MTX	0.797	0.445, 1.425	1.000
Recurrent MACE	Biologic vs. CSA	5.906	1.208, 28.874	0.019
	Biologic vs. MTX	2.937	0.520, 16.578	0.603
	Biologic vs. Phototherapy	6.165	1.268, 29.977	0.014
	CSA vs. Phototherapy	1.044	0.533, 2.044	1.000
	MTX vs. Phototherapy	2.099	0.789, 5.580	0.273
	CSA vs. MTX	0.497	0.188, 1.317	0.350

*Note:* Multivariable logistic regression model was performed with adjustment for age, sex, insurance type, concomitant psoriatic arthritis, type of medical center, and number of cardiovascular risk factors.

Abbreviations: CSA, cyclosporine; MACE, major adverse cardiovascular event; MTX, methotrexate.

^a^ 95% confidence intervals and *p*‐values were Bonferroni corrected for multiple comparisons.

## 4 Discussion

In this retrospective cohort study of patients with plaque psoriasis who experienced MACE while receiving systemic therapy or phototherapy, biologic therapies were associated with the highest treatment persistence after MACE compared with both non‐biologic systemic therapy and phototherapy. These findings were consistent in the subgroup analyses stratified by MACE history. Moreover, treatment switching following MACE was infrequent across all groups, reflecting a general preference for maintaining or discontinuing existing therapy rather than starting a new regimen.

Notably, our analysis revealed that nearly half of the patients receiving non‐biologic or phototherapy discontinued treatment following a cardiovascular event. This observation highlights a concerning gap between scientific evidence and real‐world practice. Despite psoriasis being an established independent risk factor for CVD [19], which often necessitates ongoing management, clinicians frequently discontinue treatment in real‐world settings after MACE. Such discontinuation may reflect the clinicians' concerns regarding the potential CVD risk associated with certain psoriasis treatments. Cyclosporine, for instance, has been consistently associated with increased cardiovascular morbidity and mortality in patients with psoriasis [20, 21, 22]. In contrast, phototherapy exerts a neutral effect on CVD risk, while methotrexate and biologics have been associated with lower MACE occurrence [23]. If increased cardiovascular risk from certain non‐biologic systemic agents were a clinician's major concern, one might expect a shift toward safer alternatives such as phototherapy or biologics. However, our findings showed that treatment switching following MACE was low across all therapeutic modalities, suggesting that optimal therapeutic management may be lacking in this subgroup of patients at high risk for CVD.

In this study, the patients receiving biologic therapy demonstrated the highest treatment persistence rates. This may be partly explained by the relatively well‐established cardioprotective profile of certain biologic agents compared to other psoriasis treatments. Previous studies have reported protective roles of biologic therapies against MACEs. A cohort study demonstrated that patients treated with TNF‐α inhibitors had a significantly lower incidence of new‐onset MACE compared to those receiving topical therapies, non‐biologic systemic agents, or phototherapy [24]. Similarly, a recent retrospective cohort study demonstrated that treatment with TNF‐α inhibitors, as well as IL‐12/23, IL‐17, and IL‐23 inhibitors, is associated with a reduced risk of both new‐onset and recurrent MACE in patients with psoriasis [11]. Hence, continuation or transition to biologic therapy may be a suitable option for patients with psoriasis following MACE, given the superior cardioprotective profile of these agents.

This study has some limitations, most of which reflect the common constraints associated with the use of claims‐based data. First, our data did not include key risk factors for CVD, such as family history of CVD, smoking status, alcohol consumption, or physical activity. Although we adjusted for multiple comorbidities, the possibility of residual confounding factors remains. Second, in countries with a national health insurance or similar reimbursement system, the reimbursement criteria for biologics are generally more stringent than those for non‐biologic systemic agents or phototherapy [25, 26, 27]. In Korea, under the NHI system, stringent criteria are applied both at initiation and upon reinitiation after discontinuation. These institutional factors may encourage physicians and patients to maintain biologic therapy for as long as possible, potentially contributing to the higher treatment persistence observed in this study. Nevertheless, it is difficult to fully disentangle the effects of such policy‐related influences from clinical decision‐making in real‐world settings, particularly when using claims‐based data. However, drug‐specific attributes may also play an independent role; for example, although all biologics in Korea are subject to the same reimbursement criteria, differences in treatment persistence have been observed among agents [28]. If persistence were driven entirely by policy, such inter‐drug differences would be unlikely.

Third, the varying dosing intervals of biologics may have affected the findings. For example, biologics such as IL‐12/23 and IL‐23 inhibitors, which have longer intervals between doses, may be more prone to misclassification as discontinuation due to a delay in visits. Nevertheless, subgroup analyses stratified by dosing interval showed consistent treatment persistence across biologic subtypes (data not shown).

Finally, the absence of data on psoriasis severity measures, such as the Psoriasis Area and Severity Index or body surface area involvement, limited our ability to determine whether treatment discontinuation was related to disease remission or to assess the severity and extent of any recurrence after discontinuation. This limitation also raises the possibility that patients in the biologic group had higher baseline disease severity than those in the other groups. However, most previous studies have found little or no association between baseline severity prior to treatment initiation and treatment persistence; in studies where an association was observed, higher baseline severity was generally associated with lower persistence [29, 30, 31, 32]. Therefore, this potential difference is unlikely to substantially affect the validity of our findings.

A key strength of this study is its use of comprehensive national claims data and the inclusion of a large, representative patient population. To the best of our knowledge, this is the first study to systematically evaluate treatment persistence across biologics, non‐biologic systemic agents, and phototherapy in patients with psoriasis following MACE. This gap in the literature poses challenges in optimizing post‐MACE care for this high‐risk population. By demonstrating significantly higher treatment persistence with biologics, regardless of previous MACE history, our study provides timely, real‐world evidence to guide clinical decision‐making.

In conclusion, biologic therapy was associated with significantly higher treatment persistence following MACE compared to non‐biologic systemic agents and phototherapy. Notably, despite psoriasis being a well‐established independent risk factor for CVD, physicians tend to either maintain current therapy or discontinue treatment rather than switching to potentially safer alternatives that could actively manage cardiovascular risk. These findings support the preferential continuation of biologic therapy in the post‐MACE setting and highlight the need to consider cardiovascular safety in the long‐term management of psoriasis. Incorporating these insights into future clinical guidelines and shared decision‐making processes may help optimize outcomes for patients with both dermatological and cardiovascular comorbidities.

## Ethics Statement

Approval of the research protocol by the Institutional Review Board: this study was exempted from institutional review board approval (IRB no. 07‐2022‐24).

## Consent

The requirement for informed consent was waived because only deidentified data were used.

## Conflicts of Interest

The authors declare no conflicts of interest.

## Data Availability

The data that support the findings of this study are available from the Health Insurance Review and Assessment Service (HIRA), but restrictions apply to the availability of these data, which were used under license for the current study, and so are not publicly available. Data are, however, available from the authors upon reasonable request and with permission of HIRA.

## References

[1] L. Li , K. W. Hagberg , M. Peng , K. Shah , M. Paris , and S. Jick , “Rates of Cardiovascular Disease and Major Adverse Cardiovascular Events in Patients With Psoriatic Arthritis Compared to Patients Without Psoriatic Arthritis,” Journal of Clinical Rheumatology 21, no. 8 (2015): 405–410, 10.1097/rhu.0000000000000306. 26406567 PMC4654263

[2] E. J. Samarasekera , J. M. Neilson , R. B. Warren , J. Parnham , and C. H. Smith , “Incidence of Cardiovascular Disease in Individuals With Psoriasis: A Systematic Review and Meta‐Analysis,” Journal of Investigative Dermatology 133, no. 10 (2013): 2340–2346, 10.1038/jid.2013.149. 23528816

[3] L. Liu , S. Cui , M. Liu , X. Huo , G. Zhang , and N. Wang , “Psoriasis Increased the Risk of Adverse Cardiovascular Outcomes: A New Systematic Review and Meta‐Analysis of Cohort Study,” Frontiers in Cardiovascular Medicine 9 (2022): 829709, 10.3389/fcvm.2022.829709. 35402553 PMC8990932

[4] E. J. Armstrong , C. T. Harskamp , and A. W. Armstrong , “Psoriasis and Major Adverse Cardiovascular Events: A Systematic Review and Meta‐Analysis of Observational Studies,” Journal of the American Heart Association 2, no. 2 (2013): e000062, 10.1161/jaha.113.000062. 23557749 PMC3647278

[5] W. H. Boehncke , S. Boehncke , A. M. Tobin , and B. Kirby , “The ‘Psoriatic March’: A Concept of How Severe Psoriasis May Drive Cardiovascular Comorbidity,” Experimental Dermatology 20, no. 4 (2011): 303–307, 10.1111/j.1600-0625.2011.01261.x. 21410760

[6] A. Arida , A. D. Protogerou , G. D. Kitas , and P. P. Sfikakis , “Systemic Inflammatory Response and Atherosclerosis: The Paradigm of Chronic Inflammatory Rheumatic Diseases,” International Journal of Molecular Sciences 19, no. 7 (2018): 1890, 10.3390/ijms19071890. 29954107 PMC6073407

[7] M. H. Tsai , C. Chan , M. S. Lee , and M. S. Lai , “Cardiovascular Risk Associated With Methotrexate Versus Retinoids in Patients With Psoriasis: A Nationwide Taiwanese Cohort Study,” Clinical Epidemiology 13 (2021): 693–705, 10.2147/clep.S305126. 34408498 PMC8364829

[8] Y. A. Elnabawi , A. K. Dey , A. Goyal , et al., “Coronary Artery Plaque Characteristics and Treatment With Biologic Therapy in Severe Psoriasis: Results From a Prospective Observational Study,” Cardiovascular Research 115, no. 4 (2019): 721–728, 10.1093/cvr/cvz009. 30721933 PMC6432047

[9] J. J. Wu , A. A. Joshi , S. P. Reddy , et al., “Anti‐Inflammatory Therapy With Tumour Necrosis Factor Inhibitors Is Associated With Reduced Risk of Major Adverse Cardiovascular Events in Psoriasis,” Journal of the European Academy of Dermatology and Venereology 32, no. 8 (2018): 1320–1326, 10.1111/jdv.14951. 29573294

[10] Y. A. Elnabawi , E. K. Oikonomou , A. K. Dey , et al., “Association of Biologic Therapy With Coronary Inflammation in Patients With Psoriasis as Assessed by Perivascular Fat Attenuation Index,” JAMA Cardiology 4, no. 9 (2019): 885–891, 10.1001/jamacardio.2019.2589. 31365032 PMC6669789

[11] W. J. Song , S. Oh , and H. S. Yoon , “Association Between Biologic and Nonbiologic Systemic Therapy for Psoriasis and Psoriatic Arthritis and the Risk of New‐Onset and Recurrent Major Adverse Cardiovascular Events: A Retrospective Cohort Study,” Journal of the American Academy of Dermatology 93 (2025): 141–149, 10.1016/j.jaad.2025.03.055. 40154667

[12] G. E. Fragoulis , S. Soulaidopoulos , P. P. Sfikakis , T. Dimitroulas , and D. Kitas , “Effect of Biologics on Cardiovascular Inflammation: Mechanistic Insights and Risk Reduction,” Journal of Inflammation Research 14 (2021): 1915–1931, 10.2147/jir.S282691. 34017189 PMC8131071

[13] A. Ajoolabady , D. Pratico , L. Lin , et al., “Inflammation in Atherosclerosis: Pathophysiology and Mechanisms,” Cell Death & Disease 15, no. 11 (2024): 817, 10.1038/s41419-024-07166-8. 39528464 PMC11555284

[14] G. Orlando , B. Molon , A. Viola , M. Alaibac , R. Angioni , and S. Piaserico , “Psoriasis and Cardiovascular Diseases: An Immune‐Mediated Cross Talk?,” Frontiers in Immunology 13 (2022): 868277, 10.3389/fimmu.2022.868277. 35686132 PMC9170986

[15] A. Egeberg , L. Skov , A. A. Joshi , et al., “The Relationship Between Duration of Psoriasis, Vascular Inflammation, and Cardiovascular Events,” Journal of the American Academy of Dermatology 77, no. 4 (2017): 650–656.e653, 10.1016/j.jaad.2017.06.028. 28826925 PMC5657544

[16] H. Maradit‐Kremers , M. Icen , F. C. Ernste , R. A. Dierkhising , and M. T. McEvoy , “Disease Severity and Therapy as Predictors of Cardiovascular Risk in Psoriasis: A Population‐Based Cohort Study,” Journal of the European Academy of Dermatology and Venereology 26, no. 3 (2012): 336–343. 22339785 10.1111/j.1468-3083.2011.04071.x PMC3312806

[17] J. A. Kim , S. Yoon , L. Y. Kim , and D. S. Kim , “Towards Actualizing the Value Potential of Korea Health Insurance Review and Assessment (HIRA) Data as a Resource for Health Research: Strengths, Limitations, Applications, and Strategies for Optimal Use of HIRA Data,” Journal of Korean Medical Science 32, no. 5 (2017): 718–728, 10.3346/jkms.2017.32.5.718. 28378543 PMC5383602

[18] E. Bosco , L. Hsueh , K. W. McConeghy , S. Gravenstein , and E. Saade , “Major Adverse Cardiovascular Event Definitions Used in Observational Analysis of Administrative Databases: A Systematic Review,” BMC Medical Research Methodology 21, no. 1 (2021): 241, 10.1186/s12874-021-01440-5. 34742250 PMC8571870

[19] N. Gao , M. Kong , X. Li , et al., “The Association Between Psoriasis and Risk of Cardiovascular Disease: A Mendelian Randomization Analysis,” Frontiers in Immunology 13 (2022): 918224, 10.3389/fimmu.2022.918224. 35844511 PMC9278135

[20] J. R. Hong , H. Jeong , H. Kim , et al., “The Potential Impact of Systemic Anti‐Inflammatory Therapies in Psoriasis on Major Adverse Cardiovascular Events: A Korean Nationwide Cohort Study,” Scientific Reports 11, no. 1 (2021): 8588, 10.1038/s41598-021-87766-y. 33883587 PMC8060423

[21] W. J. Choi , E. J. Park , I. H. Kwon , K. H. Kim , and K. J. Kim , “Association Between Psoriasis and Cardiovascular Risk Factors in Korean Patients,” Annals of Dermatology 22, no. 3 (2010): 300–306, 10.5021/ad.2010.22.3.300. 20711266 PMC2917683

[22] J. R. Curtis , M. I. Danila , L. Chen , et al., “Risk of Cardiovascular Outcomes Among Psoriasis Patients Treated With Biologics and Other Systemic Agents,” Journal of Psoriasis and Psoriatic Arthritis 1, no. 3 (2016): 128–137, 10.1177/247553031600100307. 29423460 PMC5800414

[23] J. Hugh , A. S. Van Voorhees , R. I. Nijhawan , et al., “From the Medical Board of the National Psoriasis Foundation: The Risk of Cardiovascular Disease in Individuals With Psoriasis and the Potential Impact of Current Therapies,” Journal of the American Academy of Dermatology 70, no. 1 (2014): 168–177, 10.1016/j.jaad.2013.09.020. 24184141

[24] J. J. Wu , M. Sundaram , M. Cloutier , et al., “The Risk of Cardiovascular Events in Psoriasis Patients Treated With Tumor Necrosis Factor‐Α Inhibitors Versus Phototherapy: An Observational Cohort Study,” Journal of the American Academy of Dermatology 79, no. 1 (2018): 60–68, 10.1016/j.jaad.2018.02.050. 29499292

[25] S. W. Youn , T. F. Tsai , C. Theng , et al., “The Marcopolo Study of Ustekinumab Utilization and Efficacy in a Real‐World Setting: Treatment of Patients With Plaque Psoriasis in Asia‐Pacific Countries,” Annals of Dermatology 28, no. 2 (2016): 222–231, 10.5021/ad.2016.28.2.222. 27081271 PMC4828387

[26] L. Puig , T. Fan , Q. Ding , and N. E. Smith , “Predictors of Biologic Treatment of Psoriasis: A Non‐Interventional Study,” ClinicoEconomics and Outcomes Research 6 (2014): 93–100, 10.2147/ceor.S54797. 24600238 PMC3933358

[27] T. Raine , M. A. Gkini , P. M. Irving , et al., “Maintaining Clinical Freedom Whilst Achieving Value in Biologics Prescribing: An Integrated Cross‐Specialty Consensus of UK Dermatologists, Rheumatologists and Gastroenterologists,” BioDrugs 35, no. 2 (2021): 187–199, 10.1007/s40259-020-00464-5. 33635522 PMC7952361

[28] S. Oh , S. Choi , and H. S. Yoon , “Available Alternative Biologics and Disease Groups Influence Biologic Drug Survival in Patients With Psoriasis and Psoriatic Arthritis,” Annals of Dermatology 34, no. 5 (2022): 321–330, 10.5021/ad.22.003. 36198623 PMC9561298

[29] E. Daudén , G. P. G. de Lima , S. Armesto , et al., “Multicenter Retrospective Study of Secukinumab Drug Survival in Psoriasis Patients in a Daily Practice Setting: A Long‐Term Experience in Spain,” Dermatology and Therapy 11, no. 6 (2021): 2207–2215, 10.1007/s13555-021-00606-9. 34561788 PMC8611164

[30] C. D. B. Elgaard , L. Iversen , and K. F. Hjuler , “Single‐Centre Real‐World Study on Drug Survival and Effectiveness of Brodalumab for Treatment of Psoriasis and Psoriatic Arthritis,” Drugs in R&D 23, no. 2 (2023): 155–163, 10.1007/s40268-023-00422-w. 37155121 PMC10293129

[31] M. E. Otero , J. M. van den Reek , M. M. Seyger , P. C. van de Kerkhof , W. Kievit , and E. M. de Jong , “Determinants for Drug Survival of Methotrexate in Patients With Psoriasis, Split According to Different Reasons for Discontinuation: Results of the Prospective Mtx‐Capture,” British Journal of Dermatology 177, no. 2 (2017): 497–504, 10.1111/bjd.15305. 28078672

[32] N. Manuelpillai , J. Armstrong , F. F. Ismail , et al., “Real‐World Guselkumab Response and Drug Survival in Australian Patients With Psoriasis: Results From the Australasian Psoriasis Registry,” Dermatologic Therapy 2024, no. 1 (2024): 8724445, 10.1155/dth/8724445.

